# Implementing an electronic public health record for policy planning in the UK military sector: Validation of a secure hashing algorithm

**DOI:** 10.1016/j.heliyon.2023.e16116

**Published:** 2023-05-19

**Authors:** Marco Tomietto, Andrew McGill, Matthew D. Kiernan

**Affiliations:** Department of Nursing, Midwifery and Health, Faculty of Health and Life Sciences, Northumbria University, Newcastle upon Tyne, United Kingdom

**Keywords:** Secure Hashing Algorithm, Military veterans, Military families, Unique identifier, Big data, Data aggregation, Data confidentiality

## Abstract

The digitalisation of healthcare services is a major resource to inform policy-makers. However, the availability of data and the establishment of a data flow present new issues to address, such as data anonymisation, records' reliability, and data linkage. The veterans' population in the UK presents complex needs and many organisations provide social and healthcare support, but their databases are not linked or aggregated to provide a comprehensive overview of service planning. This study aims to test the sensitivity and specificity of a Secure Hashing Algorithm to generate a unique anonymous identifier for data linkage across different organisations in the veterans' population. A Secure Hashing Algorithm was performed by considering two input variables from two different datasets. The uniqueness of the identifier was compared against the single personal key adopted as a current standard identifier. Chi-square, sensitivity, and specificity were calculated. The results demonstrated that the unique identifier generated by the Secure Hashing Algorithm detected more unique records when compared to the current gold standard. The identifier demonstrated optimal sensitivity and specificity and it allowed an enhanced data linkage between different datasets. The adoption of a Secure Hashing Algorithm improved the uniqueness of records. Moreover, it ensured data anonymity by transforming personal information into an encrypted identifier. This approach is beneficial for big data management and for creating an aggregated system for linking different organisations and, in this way, for providing a more comprehensive overview of healthcare provision and the foundation for precision public health strategies.

## 1 Introduction

The digitalisation of healthcare services is a major resource to inform policy-makers. However, the availability of data and the establishment of a data flow present new issues to address, such as big data management, in order to properly transform data into useful information for healthcare- and social-services planning [1].

The implementation of Electronic Health Records (EHRs) is key to providing quality and cost-effective healthcare provision. Moreover, it is essential for integrated care and to provide a comprehensive health record system, which follows individuals along their lifespan and along their contacts with different healthcare services and professionals [2,3].

The benefits of an EHR include administrative efficiency, improved access to information by healthcare professionals, patients, researchers, decision-makers, increased patient safety, and costs reduction [3]. Along with these benefits, there are also three major barriers to consider for the implementation of an EHR. At the organisational level (1), the cost of implementation is an initial bottleneck, which affects the healthcare systems worldwide in adopting an EHR. While the benefits over time are evident, the investment for implementation is not always affordable. Also, at the human resource management level (3), there is a need for trained professionals to develop and maintain the EHR [4]. Moreover, it is also necessary to plan the healthcare professionals' education to properly adopt the EHR and to overcome the resistance to change [5]. A third major barrier is related to privacy and security concerns (3). This aspect is strictly related to national and international regulations, and it needs to consider specific solutions to protect individual data [6]. This last barrier is a key issue to consider in future investments in EHRs and in human resources education and training.

### 1.1 Background and significance

Within the UK, it is reported that there are 2.4 million veterans [7]. Each year in the UK, approximately 14,000 men and women leave the armed forces and move back to civilian life [8] and a proportion of those will experience social and healthcare needs such as mental and physical health issues, homelessness and substances misuse [9].

When considering the veterans' services, data exists in three distinctive areas: government data (1) (such as data within the Department of Work and Pensions, Veterans UK or the Ministry of Justice) National Health Service Data (2) and data held by the Military Charities (3). Military Charities play a significant role in the provision of health and welfare services for former military personnel [10,11]. In 2017, it was reported that there were 278 military charities, 283 service funds (charities that provide financial funds to veterans), 82 military associations, and 65 mixed types of charities that provide support to the armed forces community [12]. Each of these charities within this sector collects data on how veterans use those services, and many veterans who need assistance will engage with one or more of those services over time. This multitude of different data flows that exist within a sector that provides significant care and welfare resources to this population creates an enormously complex, unintelligible picture of how those services are used by the veterans' population.

Despite the large population and the wide network of services and charities addressing the needs of veterans, there is a lack of summary indicators of complexity, and a lack of coordination between different organisations. Each service and charity have their own record system where different data flows currently exist. Harmonising and aggregating these data flows is crucial to develop a clear overview of the complexity of needs and to improving the social and healthcare services at the National level [12].

There is a significant need to harmonize these data flows at a population level to understanding the characteristics of those using services, where those services are being accessed and what needs are being met by those services. A harmonised or aggregated dataset such as this would create a national resource that would provide an evidence base for policy, resource allocation and service planning.

This situation is similar to the current status of epidemiological research in which researchers need to merge different datasets and develop different linkage techniques to create a comprehensive dataset for research and for informing policy-makers.

In recent years, the development of advanced cryptography techniques such as Secure Hashing Algorithms (SHAs) allowed researchers to generate a unique identifier starting from a set of variables, which should identify a unique pattern. In this way, by including a reasonable number of variables to identify a unique pattern, it is possible to generate an anonymous unique identifier across different datasets and merge them [13].

The main point to address and verify is the uniqueness of the identifier generated by the SHA and to determine if the set of variables to generate it are wide and specific enough to ensure this uniqueness.

This approach has the advantage to overcome the privacy and security concerns of an EHR and the potential to ensure a valid and reliable way to merge different datasets or dataflows from different sources over time.

### 1.2 Aims of this study

This study aims to test a SHA to generate an anonymous unique identifier for merging multiple datasets from the Military Charities Sector in the UK. Specifically, this study aims to:

1. Test the uniqueness, sensitivity and specificity of a unique identifier generated by a SHA;
2. Test data linkage by adopting the SHA-generated unique identifier.

The SHA unique identifier will be tested against the current standard identifier (military service number).

## 2 Materials and methods

### 2.1 Study design

This study adopted a retrospective design by considering two datasets from two charities in the UK. One charity provided data from two years (2021 and 2022). In this way, this study also tested both the uniqueness of the SHA-generated unique identifier in each charity and the data linkage between two different years in the same charity.

### 2.2 Secure Hashing Algorithm

The SHA is a cryptography hash function ensuring both data integrity and authentication through a digital certificate. A hash function considers an arbitrary input (e.g. a set of variables) and generates a fixed “fingerprint” string. The uniqueness of this fingerprint depends on the input. SHAs are a family of hash functions designed for the abovementioned purpose [14].

In this study, an encryption tool has been adopted and customized to the specific variables and datasets of the Military Charities Sector in the UK. The package for generating the SHA unique identifier is an open-source package available at: https://github.com/ClinicalLaboratory/Generating-Unique-IDs-from-Pateint-identification-Data-Using-Security-Models implemented in R Studio [15].

### 2.3 Data sources

Two data sets were used from two large UK Military Charities. In detail, two years of data from the Army Benevolent Fund-The Soldiers Charity (ABF 2021 and ABF 2022) and one data set from the Royal Naval Benevolent Trust (RNBT). The ABF offers support in independent living, elderly care, education and employability, mental fitness, families and housing to those that have served in the British Army. The RNBT provides financial assistance and support to those that have served in the Royal Navy, and their families. As ABF only provides support to those that have served in the British Army and RNBT support to those that have served in the Royal Navy, the SHA should not identify an individual receiving assistance from both charities, as it is expected that veterans will be supported by their single service charity only.

### 2.4 Statistical analyses

Data preparation was performed in Excel and datasets were converted in .csv format to be imported into R Studio. The SHA unique identifiers were generated by adopting R Studio and the package mentioned above [15].

Datasets were prepared by recording gender as “M” or “F”. Military service numbers have been checked for consistency of the format (e.g. upper or lower cases, sequence of letters and numbers expected). Missing data in one or both variables considered for the SHA generation (gender and military service number) have been deleted. The unique identifiers were generated by using the same set of input variables (gender and military service number) for the SHA.

In step 1, descriptive statistics have been performed to compare the frequency of unique records between the SHA-generated unique identifiers and the current gold standard. Chi-Square test was performed to compare those frequencies (p < 0.05). Sensitivity and specificity of the SHA unique identifiers were calculated against the current gold standard (military service number) to detect the SHA ability to correctly identify a unique record (sensitivity) and to correctly reject a duplicate record (specificity). The ROC (Receiver Operator Characteristics) curve was plotted and the area under the curve was calculated. The SHA unique identifiers, sensitivity, specificity, chi-square and the ROC curve were analyzed using SPSS v28 for Windows [16].

In step 2, data linkage between the datasets and their records was compared by considering the two identifiers and by adopting a one-to-one procedure in Stata v13 [17].

**Fig. 1:**
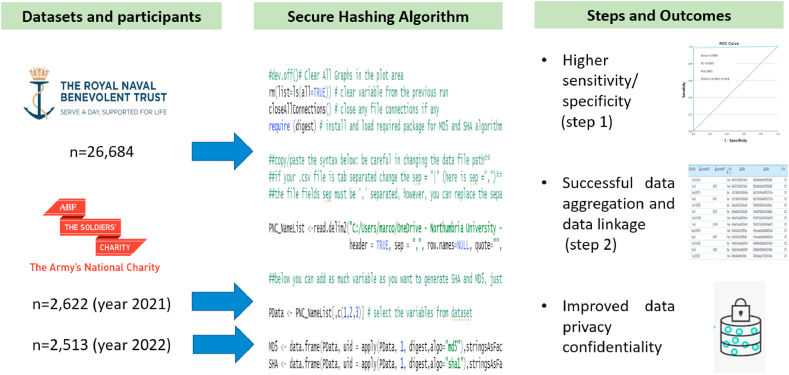
Data sources, steps for data analysis and main outcomes.

Fig. 1 presents the data sources, the steps for data analysis and the main outcomes of this study.

### 2.5 Ethical considerations

This research complies with the Data Protection Act (2018), which includes the General Data Protection Regulations [18]. Ethical approval has been provided by Northumbria University (reference number: 2055).

## 3 Results

The dataset from ABF 2021 had 2622 records suitable for SHA generation, the ABF from 2022 dataset 2513 records, and the RNBT dataset 26,684.

In each dataset primary (unique) and duplicate cases were identified by military service numbers and SHA unique identifiers for comparing the rate of unique records detected by the SHA and for calculating sensitivity and specificity.

**Fig. 2:**
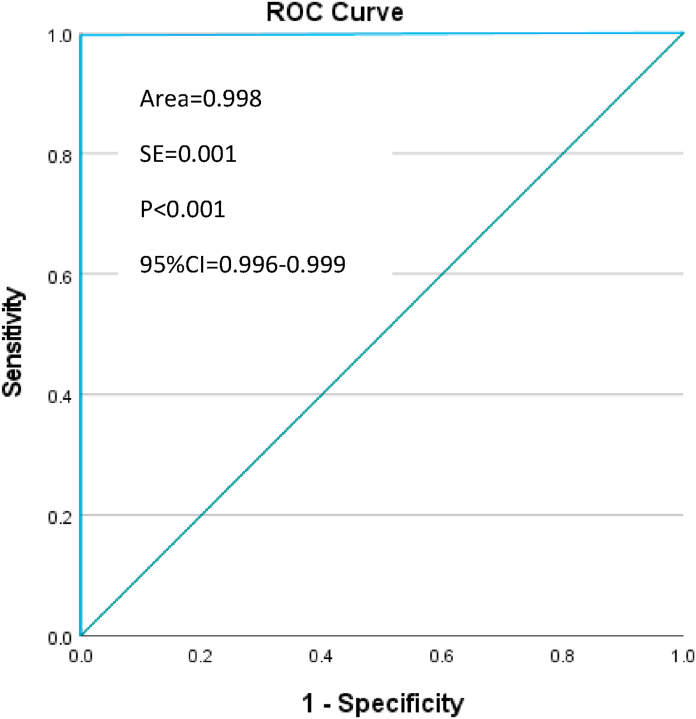
ABF 2021 dataset. ROC curve of the SHA identifier against the gold standard. (For interpretation of the references to colour in this figure legend, the reader is referred to the Web version of this article.)

In the ABF 2021 dataset, 2296/2622 primary cases (87.6%) were identified by military service number and 2306/2622 (87.9%) by SHA unique identifier. The rate of primary cases identified by the SHA algorithm, compared to those identified by the military service number, was statistically significant (chi-square = 2530.549, p < 0.001) with a sensitivity of 100% (95%CI = 99.8%–100%) and a specificity of 96.9% (95%CI = 94.4%–98.5%) (Table 1). The area under the ROC curve was 99.8% (95%CI = 99.6–99.9, p < 0.001) (Fig. 2).

**Table 1:** ABF 2021 dataset. Cross-tabulation military service number and SHA identifier.

		Military Service Number			
		Primary cases	Duplicate cases	Total	
SHA identifier	Primary cases	2296 (100%*)	10	2306	χ^2^ = 2530.549 p < 0.001
	Duplicate cases	0	316 (96.9%**)	316	
	Total	2296	326	2622	

*sensitivity; **specificity.

**Fig. 3:**
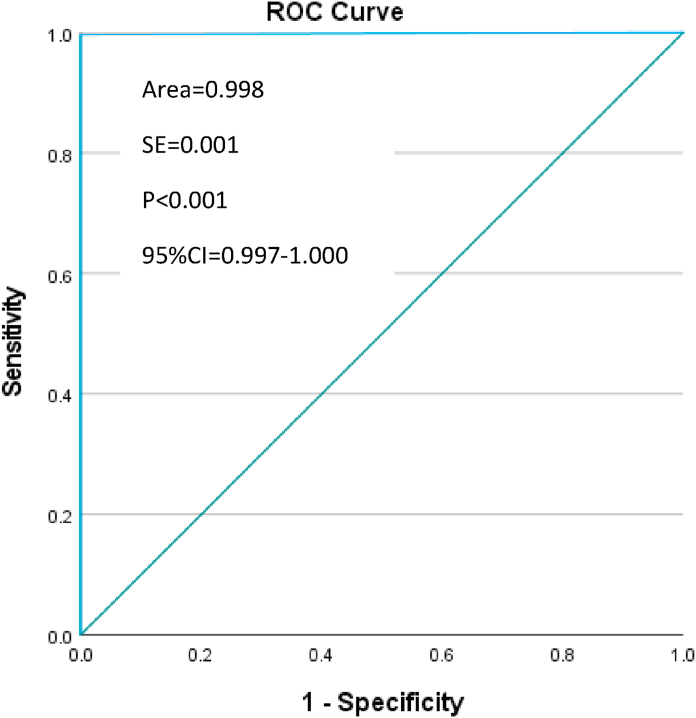
ABF 2022 dataset. ROC curve of the SHA identifier against the gold standard. (For interpretation of the references to colour in this figure legend, the reader is referred to the Web version of this article.)

In the ABF 2022 dataset, 2344/2513 primary cases (93.3%) were identified by military service number and 2353/2513 (93.6%) by SHA unique identifier. The rate of primary cases identified by the SHA algorithm was significantly associated with the standard approach (chi-square = 2370.071, p < 0.001) with a sensitivity of 100% (95%CI = 99.8%–100%) and a specificity of 94.7% (95%CI = 90.1%–97.5%) (Table 2). The area under the ROC curve was 99.8% (95%CI = 99.7–100, p < 0.001) (Fig. 3).

**Table 2:** ABF 2022 dataset. Cross-tabulation military service number and SHA identifier.

		Military Service Number			
		Primary cases	Duplicate cases	Total	
SHA identifier	Primary cases	2344 (100%*)	9	2353	χ^2^ = 2370.071 p < 0.001
	Duplicate cases	0	160 (94.7%**)	160	
	Total	2344	169	2513	

*sensitivity; **specificity.

**Fig. 4:**
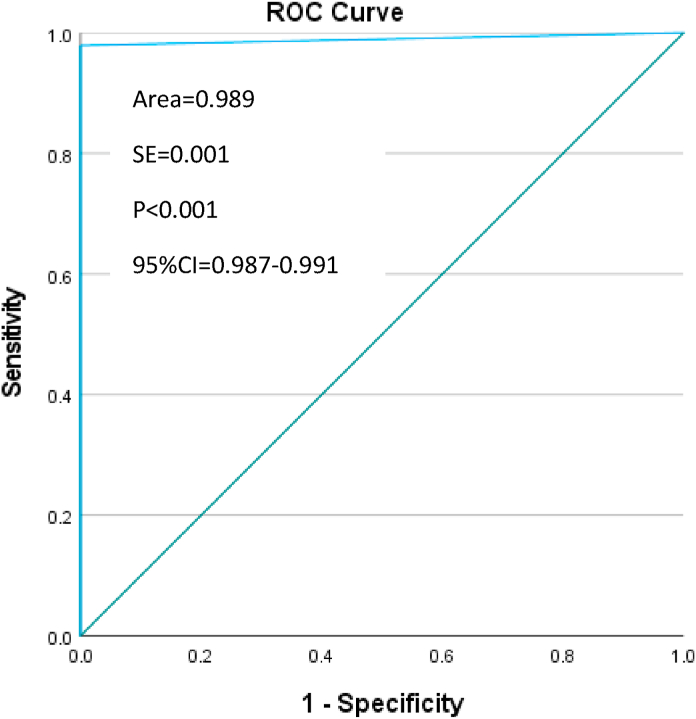
RNBT dataset. ROC curve of the SHA identifier against the gold standard. (For interpretation of the references to colour in this figure legend, the reader is referred to the Web version of this article.)

In the RNBT dataset, 6601/26,684 primary cases (24.7%) were identified by military service number and 6743/26,684 (25.3%) by SHA unique identifier. In total 142 primary cases were additionally identified by the SHA (chi-square = 25947.662, p < 0.001), with a sensitivity of 100% (95%CI = 99.9%–100%) and a specificity of 99.3% (95%CI = 99.2%–99.4%) (Table 3). The ROC curve supports the optimal sensitivity and specificity of the SHA-generated unique identifiers compared to the gold standard, the area under the curve was 98.9% (95%CI = 98.7–99.1, p < 0.001) (Fig. 4).

**Table 3:** RNBT dataset. Cross-tabulation military service number and SHA identifier.

		Military Service Number			
		Primary cases	Duplicate cases	Total	
SHA identifier	Primary cases	6601 (100%*)	142	6743	χ^2^ = 25937.365 p < 0.001
	Duplicate cases	0	19,941 (99.3%**)	19,941	
	Total	6601	20,083	26,684	

*sensitivity; **specificity.

A screening of the primary records detected by the SHA compared to those ones detected by the military service number across the two datasets revealed that the same military service number was associated with different individuals (meaning a different pattern of variables). In this way, it is possible to confirm that the identifier generated by the SHA was able to identify more accurately the uniqueness of each individual.

In step 2, data linkage was tested. The RNBT dataset was matched with the ABF 2021 and the ABF 2022 datasets by the SHA unique identifiers: as expected, no match was found between the datasets. Similarly, the ABF 2021 dataset and the ABF 2022 dataset were matched by the SHA unique identifiers, and 1771 records were matched by the SHA unique identifier which would indicate that 1771 individuals were receiving care from the charity over two years. Due to the lower sensitivity of the military service number, it was not possible to match the records from the two ABF datasets by military service number.

## 4 Discussion

Ensuring the privacy and security of an EHR is a complex issue that includes solutions at different levels: physical, administrative, and technical [19]. At the physical level, it is necessary to focus on network security from the workstations and servers to WiFi radio frequencies and physical access to the devices in the network. From an administrative perspective, it is necessary to establish a risk management system, identify a chain of responsibilities for the system security evaluation, and plan a recovery strategy. The technical level considers users' authentication, virus checking, firewall protection, and data encryption [19]. This paper focused on this last aspect to link different datasets and dataflows in a valid and reliable way and, at the same time, to protect data confidentiality by ensuring data anonymisation. This paper described a procedure to ensure the uniqueness of records' identification by adopting a SHA. This approach demonstrated a better performance in improving the identifiers' uniqueness, compared to the current standard. Moreover, the SHA generated unique identifier allowed the anonymisation of data by deleting the individuals' personal information once the unique identifier is generated. The SHA unique identifier also demonstrated an improved data linkage.

Previous approaches for matching records have shown controversial levels of accuracy [20]. A sufficient number of input variables is crucial to ensure the uniqueness of an identifier and accurate records' matching. The SHA tested in this paper demonstrated an optimal sensitivity and specificity and it also performed better than the previous approach when matching different datasets. Moreover, the sensitivity of the SHA unique identifiers is optimal in the 3 datasets and the specificity improved with the sample size. This supports the adoption of the unique identifier generated by the SHA, as it is able to improve the records' uniqueness, data anonymisation and data linkage, especially when managing large datasets and big data [13].

Furthermore, this approach allows data to be aggregated from different organisations. This enables the interactions between veterans and military charities to be observed at the population level, irrespective of which arm of the British military Forces they served in, and in turn, representing their social and health needs. This is a fundamental step to start planning healthcare provision at the National level and addressing the complex needs of this population by preventing poor health and social conditions, promoting healthy behaviours, and early detection of conditions or situations which might impact severely on the service users. The role of big data management in public health and the military veterans space is crucial to allocate the resources to promote healthy populations [21] and the implementation of unique and anonymous identifiers through a SHA is a promising solution to develop reliable data aggregation from different sources. Furthermore, this approach will help develop the implementation of health and social care strategies for veterans, and it will contribute to a more precise analysis of individuals and groups at the population level, leading to improved public health interventions for veterans [22]. While the benefit of public health is clear, it is necessary to consider also the risks related to this approach, as this level of detail is a potential harm for the privacy and security of individuals. However, it is argued that the major advantage of the SHA is that it enables both data aggregation and precision public health, but also ensures data anonymity and confidentiality [19]. The SHA and the generation of a reliable and anonymous unique identifier is the gateway for data aggregation, and, over time, implementing big data in the military veterans' population. This then has the potential to then develop machine learning and artificial intelligence solution for precision public health [23,24].

## 5 Future implementation

The SHA approach is recognised as the gold standard for generating a unique identifier. It requires good-enough computing capability to be performed, especially for large datasets [13]. If the computing capability is not a reason of concern, this approach is recommended.

In some cases, the SHA can generate the same identifier for different records due to data entry flaws, which could lead to the same input variables for different records. For this reason, data checking and preparation before performing the SHA is essential to ensure the unique identifier’s accuracy [25].

In this study, the largest dataset included 26,684 records. While this sample size was adequate for methodologically testing the algorithm, further tests on big data are recommended. Moreover, the SHA has been performed by considering two input variables to generate the unique identifier. While the results are satisfactory, it is recommended to use more variables to better ensure the identifiers' uniqueness.

## 6 Conclusion

Veterans are a considerable population in the UK and they present with complex needs at the social and public health level. Military Charities are key in addressing this complexity and in providing support to this population. However, they act as separate organisations and different datasets, and information flows exist without being aggregated or providing a comprehensive overview of how those services are used by the veterans' population. This study implemented a SHA for generating a unique identifier to facilitate data aggregation and linkage, so as to aggregate these datasets and support a better healthcare provision at the public health level. This technical solution also addresses data confidentiality and anonymisation by protecting personal data while ensuring accurate data aggregation.

## Authors contributions

Marco Tomietto: Wrote the paper, designed the study, analyzed and interpreted data; drafted the article, and critically revised its intellectual content; approved the version submitted. Andrew McGill: Wrote the paper, acquired data, analyzed and interpreted data; drafted the article, and critically revised its intellectual content; approved the version submitted. Matthew D. Kiernan: Wrote the paper, designed the study, interpreted data; drafted the article, and critically revised its intellectual content; approved the version submitted.

## Data availability statement

The data that has been used is confidential.

## Funding

The study was funded by the Armed Forces Covenant Fund - Map of Need - Ref 3590.

## Declaration of competing interest

The authors declare that they have no known competing financial interests or personal relationships that could have appeared to influence the work reported in this paper
